# The Role of Epithelial-Derived Extracellular Vesicles in Allergic Sensitisation: A Systematic Review

**DOI:** 10.3390/ijms26125791

**Published:** 2025-06-17

**Authors:** William Browne, Georgina Hopkins, Stella Cochrane, Victoria James, David Onion, Lucy C. Fairclough

**Affiliations:** 1 School of Life Sciences, University of Nottingham, Nottingham NG7 2RD, UK; msxwb2@exmail.nottingham.ac.uk (W.B.); georgina.hopkins@nottingham.ac.uk (G.H.); david.onion@nottingham.ac.uk (D.O.); 2 Unilever SERS, Colworth Science Park, Sharnbrook MK44 1LQ, UK; stella.a.cochrane@unilever.com; 3 School of Veterinary Medicine and Science, Biodiscovery Institute, University of Nottingham, Nottingham NG7 2RD, UK; victoria.james@nottingham.ac.uk

**Keywords:** allergy, epithelial cells, extracellular vesicles, sensitisation, tolerance

## Abstract

The aim of this systematic review was to evaluate the current evidence for the involvement of epithelial-derived extracellular vesicles (EVs) in Immunoglobulin E (IgE)-mediated allergic sensitisation. Original clinical and research studies specifically examining the effect of epithelial-derived EVs in IgE-mediated allergic sensitisation were included. Non-IgE mediated allergies, abstracts and review articles were excluded. A total of 18 publications were identified from three databases (EMBASE, Web of Science and PubMed) that indicate epithelial-derived EVs have the potential to promote tolerance or allergic sensitisation. For example, epithelial-derived EVs have the potential to promote IgE-mediated allergic sensitisation by delivering mRNAs that promote T helper 2 (Th2) polarisation and cytokine secretion, or promote tolerance through the induction of T regulatory (Treg) cells. The results also indicate that the potential role of epithelial-derived EVs in IgE-mediated allergic sensitisation may be dependent on the barrier, with all publications related to intestinal epithelium driving tolerance, but publications on nasal and bronchial/alveolar epithelia gaving mixed effects. No publications were found on cutaneous epithelia. Taken together, the literature suggests that epithelial-derived EVs play a key role in influencing IgE-mediated allergic sensitisation. Further research examining all epithelial barriers, using both robust human in vitro models that give more biologically relevant information, as well as clinical studies, are required to further characterise the role of epithelial-derived EVs in IgE-mediated allergic sensitisation.

## 1. Introduction

IgE-mediated allergy, also known as Type 1 hypersensitivity or atopic allergy, is a subtype of allergy involving immunoglobulin E (IgE) and is characterised by the rapid onset of symptoms after contact with an allergen. This type of allergy has seen an increase in prevalence over the past decades, with up to an estimated 30% of adults and 40% of children now being sensitised to at least one allergen, such as pollen, dust or food [1]. In conjunction with this, there has also been an increased burden on healthcare systems [1,2]. Sensitisation is the first stage in the development of an IgE-mediated allergy. It occurs after initial allergen exposures and results in an individual’s mast cells and basophils becoming primed with IgE-specific antibodies to a given allergen. Once sensitised, individuals can progress to being symptomatic with further exposures to an allergen, leading to the cross-linking of IgE and release of chemical mediators from mast cells and basophils, which causes symptoms such as rhinitis, asthma, urticaria and, in rare cases, anaphylaxis. Proteins are the predominant triggers of allergic sensitisation and the most common target of the triggered IgE response; however, recent research has also implicated both lipids [3,4,5] and carbohydrates [6,7,8] as other molecules that can influence allergic sensitisation.

The epithelium serves as the first barrier to the external environment and allergens. Once thought of as just a physical barrier, the epithelium has now been shown to have a more complex relationship with the immune system, interacting with and helping to orchestrate immune responses through its communication with dendritic cells, effector T cells and macrophages [9]. Dysfunction and penetration of the physical barrier are frequently observed in allergic diseases, representing one of the initial steps in allergic sensitisation. Some allergens have been shown to cleave tight junctions (TJs) between cells, facilitating allergen entry and driving subsequent allergic sensitisation [9,10,11,12]. This forms the basis of the epithelial barrier hypothesis, and the penetration of these TJs has been shown to facilitate the induction and polarisation of the immune system towards a Th2 response through the secretion of cytokine mediators, such as interleukin (IL)-25, IL-33 and thymic stromal lymphopoietin (TSLP) [13,14,15,16,17]. Moreover, the route of allergen exposure is believed to influence allergic sensitisation. The dual allergen exposure hypothesis suggests that early oral exposure to peanuts promotes tolerance, while exposure through the skin, without concurrent oral exposure, leads to allergy [18]. Additionally, more recent studies have updated the initial hypothesis to also include the airway as an alternative route of sensitisation to peanut, leading to food allergy [19]. Consequently, it is now well-established that epithelial cells play a key role in IgE-mediated allergic sensitisation, though the details of the mechanisms involved are still to be fully elucidated.

Recent research has started to shed light on a new potential mediator, extracellular vesicles (EVs), secreted by epithelial cells, that could influence the development of an allergic phenotype [20]. EVs are defined as membrane-bound particles released from cells, including epithelial cells, fibroblasts, mesenchymal cells, dendritic cells, B cells, T cells, mast cells and tumour cells, among others. They have an inability to self-replicate and can serve as intercellular communicators [21]. The presence of EVs has also been shown in multiple body fluids, including saliva, plasma, breast milk, urine, bronchoalveolar lavage and malignant effusions. EVs vary significantly in size, ranging from 30 nm to 10 μm, and are currently divided into the following subtypes: microvesicles, exosomes, apoptosomes and autophagic EVs. This classification reflects their size, biogenesis, method of release and function [22,23,24,25]. EVs can facilitate intercellular communication through the transmission of various biomolecules (lipids, proteins, sugars and nucleic acids) [23,26]. The transmission of these biomolecules or “cargo” to other cells, or simply the receptor-mediated binding of EVs at the cell surface, results in the downstream influence of essential cellular processes required for the maintenance of homeostasis [27,28], with EVs being shown to influence processes such as immune surveillance [29], tissue repair [30] and blood coagulation [31].

Epithelial-derived EVs have recently been highlighted in the pathogenesis of allergies, with roles in type 1 hypersensitivity disorders, such as asthma and allergic rhinitis (AR), being reported [20,32,33,34]. Although the literature is limited, research into epithelial-derived EVs and their facilitation of immune communication could provide the potential to better understand the mechanisms that underpin allergic sensitisation. In addition, this may lead to an insight into new therapeutic strategies, as well as interventions, to help mitigate the ever-increasing prevalence of Type 1 hypersensitivities. Thus, this systematic review aims to investigate the current understanding of the potential role of epithelial-derived EVs in IgE-mediated allergic sensitisation, whether indirectly (e.g., through affecting epithelial barrier integrity) or more directly through the alteration of the immune environment (e.g., by promoting a Th2-dominant milieu).

## 2. Materials and Methods

### 2.1. Search Strategy

This review was conducted following the Preferred Reporting Item for Systematic Reviews and Meta-Analyses (PRISMA) 2020 guidelines. Relevant articles were identified using three databases: EMBASE, PubMed and Web of Science up to 30 January 2025. A search for grey literature was also conducted with no relevant articles being found. Databases were filtered for the removal of review articles as well as articles not published in English.

The search terms used, constructed around the two key terms ‘epithelial vesicles’ and ‘allergy’, were as follows: “Epithelial,” “Epithelium,” “BALF,” “ Bronchoalveolar Lavage Fluid,” “Lavage” and “Extracellular Vesicles,” “EVs,” “Microvesicle,” “Exosome,” “Ectosome,” “Shedding Vesicle,” “Microparticle” and “Allergic Response,” “Allergy,” “Allergen,” “Sensitisation,” “Type 1 Hypersensitivity,” “IgE,” and “Tolerance” using the advanced search settings for each database, as per Supplementary Materials.

**Figure 1:**
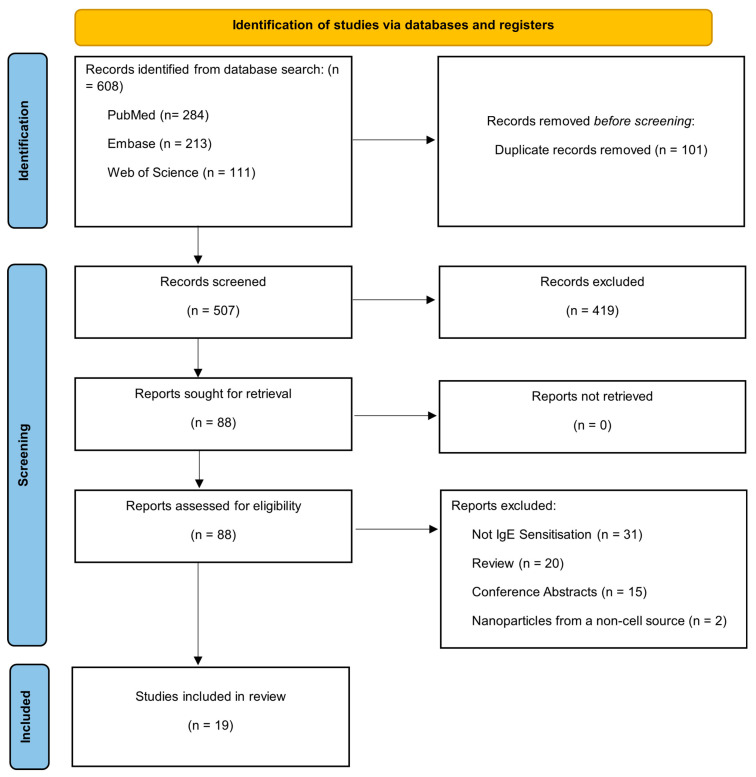
A PRISMA 2020 flow diagram summarising the process for the selection of articles within the study.

The search yielded a total of 608 articles: 284 from PubMed, 213 from Embase and 111 from Web of Science. Articles from the searches were imported into EndNote where duplicates were removed, and the remaining articles were screened initially by title and then subsequently by abstract to remove articles not relevant to the potential role of epithelial-derived EVs in IgE-mediated allergic sensitisation. This initial screening was subsequently validated by an independent reviewer. The remaining articles then had their full texts screened and were evaluated by WB, and independently by GH, for inclusion in the study using the inclusion/exclusion criteria outlined in Table 1. A PRISMA 2020 diagram outlines the process followed for the identification and inclusion of relevant articles in this systematic review (Figure 1).

### 2.2. Data Extraction and Quality Assessment

Ultimately, 18 papers were found to provide information on the potential role of epithelial-derived EVs in IgE-mediated allergic sensitisation and so were eligible for inclusion. Data regarding the design of the research was subsequently reviewed and the studies’ quality was scored based on a set of criteria outlined in Table 2. Scores were attributed to each paper within the following sections: Model Used, Allergen Characterisation, Robustness of the Model, Sample Size, EV Isolation and EV Characterisation. Due to the variety of different models utilised in the papers, it is possible for studies to receive multiple scores relating to the models, their robustness and sample size. Consequently, a paper’s final score is reflected as a percentage of the aggregate total marks for all models. The aim of this scoring system was to allow for a comprehensive evaluation of research by the current guidelines for EV research, with low scores not reflecting a study’s validity and reliability, but showing the study might lack factors that could further strengthen it.

## 3. Results

### 3.1. PRISMA and Publication Selection

**Figure 2:**
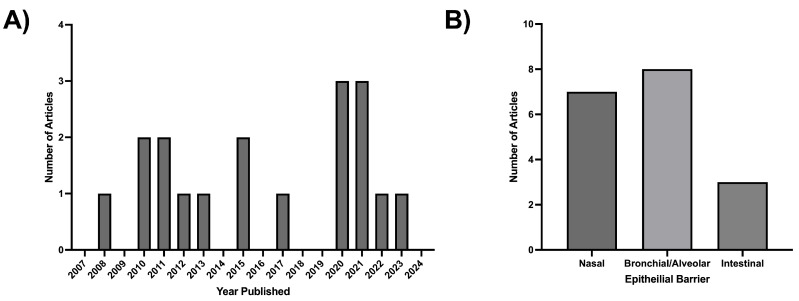
Metrics on publications identified for inclusion in the review. (**A**) Number of publications over time; (**B**) Number of publications concerning each epithelial barrier.

A total of 18 publications fulfilling the criteria for inclusion were identified in this review [35,36,37,38,39,40,41,42,43,44,45,46,47,48,49,50,51,52]. The earliest study was published in 2008 and, from this point onwards, there has been a relatively consistent publication rate (Figure 2A). The results indicate that currently, all research in this area focuses on three epithelial EV sources, namely the nasal, bronchial/alveolar and intestinal epithelia (Figure 2B). In this review, the results have been divided by these three sources of EVs, with details on their potential role in allergic sensitisation reported.

### 3.2. Nasal Epithelial-Derived Extracellular Vesicles (ne-EVs)

In total, seven publications with information on the potential role of EVs derived from the nasal epithelium in IgE-mediated allergic sensitisation were identified (Table 3) [35,36,37,38,39,40,41]. Two of the publications appeared to contain repeated data and raised questions regarding the controls and methodologies used. As a result, these are not described in detail here but are included in Table 3 as they met the search and screening criteria described [35,36]. Of the five other publications identified, four described the effects of ne-EVs on Th1/Th2 polarisation, which could influence IgE-mediated allergic sensitisation. In one paper, IL-10-secreting monocytes were shown to be induced by nasal epithelial EVs containing mRNA-146α. These IL-10+ monocytes exhibited an immunosuppressive effect on CD4+ effector T cells and subsequent Th2 polarisation [37]. In another, the examination of variation in epithelial EV cargo between healthy patients and individuals with AR revealed significant differences in the expression of a wide variety of mRNAs. Specifically, these mRNAs were involved in key pathways associated with allergic development, such as c-fos, Lyn and MUC7 [38]. An investigation into the relative expression of microRNA-146α-5p (miR-146α-5p) in the nasal epithelial-derived EVs revealed significant downregulation in AR patients. The expression of miR-146α-5p was shown to play an important role in the Th1/Th2 differentiation, with lower expression being demonstrated to promote Th2 differentiation and IL-4 cytokine secretion from naïve CD4+ T cells [41]. Two studies examined the influence of long non-coding RNA (lncRNA) cargo in ne-EVs [39,40]. Growth arrest specific 5 (GAS5) was identified as cargo of ne-EVs. GAS5 was shown to influence Th1/Th2 differentiation through the downregulation of T-bet and the enhancer of Zeste 2 polycomb repressive complex 2 subunit (EZH2), suppressing Th1 differentiation and promoting Th2 polarisation [39]. Ne-EVs containing LncRNA nuclear paraspeckle assembly transcript 1 (NEAT1) were shown to facilitate the pathogenesis of AR through the induction of IL-13-mediated inflammatory response, as well as nasal epithelial cell apoptosis. The ne-EV-induced damage to the epithelium facilitates allergen barrier penetration and ultimately allergic sensitisation [40].

With regards to quality scores, all publications reporting information related to the potential role of ne-EVs in allergic sensitisation utilised human AR patient samples. In general, all studies received good human study robustness scores, with all studies seeking allergic patients from a clinical setting [35,36,37,38,39,40,41]. Five publications also included defined healthy control patient samples [36,37,38,39,40,41]. Six studies describe using a diagnostic test such as serum-IgE measurement or skin testing to identify allergen-sensitised or allergic patients [35,36,37,38,39,41]. Only one study did not perform such a test [40]. Five studies used more than 11 participants per group, receiving maximum sample size scores [35,37,39,40,41]; however, one publication did not include a control group [35]. Two studies received lower sample size scores with one study utilising 10 participants per group [37] and the other study, whilst having more than 11 participants in their allergic groups, only had 10 controls, consequently receiving a lower score [36]. Five studies also utilised a cell line model [35,36,38,40,41], with two of these using the transformed cell line RPMI2650 [35,36,38,41] and one using primary cells [40]. The reporting on the exposure of these cell lines was generally poor and only two studies fully defined their exposure [40,41]. Two studies also implemented animal models [37,41] and received identical robustness and sizing scores, fully defining exposure and using six to ten animals per group. In terms of defining the sensitisation material used, all papers had poor scores. While not all papers used allergens in their models, those that did only reported the allergen used and provided no further information, such as protein or endotoxin measurements [35,36,38,41].

Concerning EVs, six of seven studies used sequential ultracentrifugation stages for the isolation of EVs [35,36,37,38,39,41], with one study utilising an ExoQuick EV precipitation kit [40]. EV characterisation varied between the papers; however, six of the seven studies utilised Western blots [35,36,37,39,40,41] to characterise either EV cargo [35,36] or surface proteins [37,39,40,41]. Five studies used microscopy techniques, with two using immunogold electron microscopy [35,36] and three using using conventional TEM imaging [39,40,41]. RTq-PCR was also used for cargo analysis and quantification in five papers [37,38,39,40,41]. Two studies used nanoparticle tracking analysis (NTA) to profile EV sizing [40,41]. Lastly, one paper used flow cytometry to profile tetraspanin biomarkers on EVs [38]. In general, none of the studies received a characterisation score below ‘poor’ and all made an attempt to characterise isolated EVs. Two studies received ‘poor’ characterisation scores despite using multiple complimentary techniques due to a lack of EV biomarker characterisation [35,36], and the remaining five studies all profiled at least one EV biomarker alongside complimentary techniques [37,38,39,40,41]. The characterisation scores were also observed to improve with publishing date. The lowest scores received by the studies were 52% [37] and 53% [35], with all other studies scoring 56% and above [36,38,39,40,41]. One study received a score of 76% due to its robust use of human, murine and cell line models coupled with its comprehensive EV characterisation, with this study being the only one of the seven to receive the maximum characterisation score [41]. All scores attained by these studies are fully outlined in Table 4.

### 3.3. Bronchial/Alveolar Epithelial-Derived Extracellular Vesicles (bae-EVs)

Eight publications reporting data on the potential role of bae-EVs in allergic sensitisation were identified (Table 5). Two publications investigated the influence of EVs isolated from the bronchial alveolar lavage fluid (BALF) of mice tolerised to the allergen Ole e 1 (ExoTol). The first study revealed that Exotol was able to inhibit a Th2 response through the suppression of IgE and IgG1 and upregulation of TGF-β [42]. A follow-on study investigated Exotol’s impact on sensitisation to other antigens. In this study, a ‘bystander suppression’ was observed, with Exotol inhibiting sensitisation to the allergen Bet v 1, suppressing IgE and IgG1, as well as the Th2 cytokines IL-5 and IL-13 [43]. One study demonstrated that LPS exposure enhanced bae-EVs’ production and that the LPS-induced bae-EVs enhanced sensitisation to ovalbumin in mice, as well as promoted TNF-a and IL-6 secretion in macrophages [44]. Enhanced bae-EV secretion and cargo changes were also noted in Th2 cytokine-stimulated epithelial cells in another study. These bae-EVs were subsequently shown to induce monocyte proliferation, and the suppression of their secretion was shown to alleviate asthmatic symptoms [45]. A study focussed on miRNA expression reported that there were increased amounts of bae-EVs in BALF from HDM-exposed mice compared to sham-controlled exposed mice and that the miRNA cargo was significantly changed. An analysis of the miRNA cargo suggested selectively sorting miRNA, including Th2 inhibitory miRNAs, including those with IL-13 and IL-5Ra as putative targets, into bae-EVs with HDM exposure [46]. Three further publications reported the influence of bae-EVs on DCs, with one study evaluating the miRNA cargo of EVs derived from primary normal human bronchial epithelial cells treated with IL-13 to induce an asthma-like phenotype and EVs isolated from nasal lavages of children with asthma. An analysis of the EVs from the IL-13-treated NHBE cells revealed changes in the expression of 16 miRNAs, and the miR-34a, miR-92b and miR-210 levels in the EVs from the nasal lavages correlated with lung function parameters. A subsequent pathway analysis predicted these miRNAs could help regulate Th2 polarisation and DC maturation [47]. Another study examining the effect of ovalbumin (OVA) allergen challenge on the airway epithelium of mice revealed the enhanced secretion of bae-EVs carrying OVA. These OVA-induced bae-EVs promoted the infiltration of neutrophils, monocytes and DCs into the lung, as well as induced macrophages to secrete the proinflammatory cytokines IL-6, TNF-a and IL-1β [48]. Lastly, bae-EVs secreted after HDM stimulation were shown to facilitate the recruitment of DCs in the lung and to activate DC through cargo contactin-1 (CNTN1), as well as upregulating the expression of CD40, ultimately promoting Th2 differentiation [49].

With regards to quality scores, of the eight studies looking at the influence of bae-EVs on allergic sensitisation, seven utilised mouse models [42,43,44,45,46,48,49]. The reporting of the sensitisation protocol in these models was generally good, with all seven studies clearly defining this [42,43,44,45,46,48,49]. The robustness of the models was variable, with two studies using more than 11 mice per group [42,46], one using six to ten mice per group [45] and four studies using five or fewer mice [43,44,48,49]. Five of the seven studies utilising mouse models also implemented a cell culture model [42,43,45,48,49], with three using murine primary cells [42,43,49] and two using a transformed cell line [45,48]. Only two studies utilised human patient samples, with just six to ten participants per group [47,49], and whilst both studies identified patients from a clinical setting, only one provided information regarding their allergic status, performing a skin test and measuring serum IgE [47].

Concerning EVs, five studies isolated EVs using either ultracentrifugation (UC) or serial UC [42,43,45,48,49], and one study implemented a sucrose cushion as part of UC [44]. Two studies isolated EVs using an ExoQuick precipitation kit [46,47]. EV characterisation in all the studies was suitable, with eight studies receiving a score of ‘good’ or better. All eight studies used Western blot analysis to characterize exosome biomarkers as well as TEM to confirm the presence of isolated EVs [42,43,44,45,46,47,48,49]. Four studies profiled EV cargo using RTq-PCR [46,47,48,49], with one also using a sermiR miRNA exosome profiling kit [47]. Three studies profiled sizing using NTA [47,48,49]. Lastly, two studies also used flow cytometry to analyse EV biomarker expression [42,43]. The highest-scoring paper (79% [47]) utilised two high-scoring robust model systems, as well as an ExoQuick precipitation kit for isolation, followed by comprehensive EV characterisation [47]. The lowest score received by a study was 52% [44]; this was mainly due to the use of just one model with a small sample size. All scores are outlined in Table 6.

### 3.4. Intestinal Epithelial-Derived Extracellular Vesicles (ie-EVs)

Only three studies evaluating the role of intestinal epithelial-derived EVs (ie-EVs) were identified (Table 7). All three of these studies generated data on the potential role of ie-EVs in food allergy. One study showed intestinal epithelial cells (IECs) post-OVA uptake secrete ie-EVs containing integrin αvβ6 and OVA. These αvβ6/OVA ie-EVs induced antigen-specific tolerogenic Tregs and TGF-β+ DCs, suppressing Th2 responses in the gut [50]. Another study using vasoactive intestinal peptide deficient (VIPd) and wild-type mice showed that the VIPd mice failed to induce type 1 regulatory T cells in the intestine, and that exposure to VIP in culture induced IL10 expression in IECs. Exosomes derived from OVA/VIP-primed IECs carried allergen-MHC II complexes, as well as IL-10, and these OVA/VIP-primed ie-EVs were able to induce Tr1 differentiation in OVA-specific CD4+ cells [51]. The administration of OVA/VIP-primed ie-ECs also suppressed experimental food allergy. Lastly, ie-EVs isolated from the lamina propria of OVA-fed mice, which contained OVA, were characterised and reported to have an increased expression of MHCII compared to those from naïve mice. OVA was also detected in the EVs from the OVA-fed mice. EVs from the OVA-fed mice were also reported to induce CD4+Foxp3+T cell differentiation, as well as promote the secretion of Treg-promoting cytokines IL-10 and TGF-β in macrophages [52].

With regards to quality scores, all three studies examining ie-EVs utilised mouse models [50,51,52], with two studies fully defining sensitisation protocols [50,51] and one study not disclosing the stimulation concentration, ultimately receiving a lower score [52]. Furthermore, the same study did not define its sample size [52], with the other two studies using six to ten mice per group [50,51]. In addition to the use of mouse models, two studies also used cell models [50,51]. Both models utilised received low robustness scores due to the use of immortalised cell lines; however, the definition of exposure in the models was fully detailed.

Concerning EVs, the isolation methods used in all three studies were generally ‘poor’ with all three using UC. One study, however, did use a sucrose cushion, receiving a slightly higher score [52]. The characterisation of EVs in the studies was variable and poor. Two of the three papers used microscopy to confirm the presence of isolated EVs [50,52] with one using TEM [52] and one using immunogold EM [50]. One study sized EVs using dynamic light scattering [52]. Two studies that used Western blots to profile EV biomarkers lacked suitable controls. Consequently, no study scored more than a score of ‘fair’ in terms of characterisation, despite using multiple complementary techniques. The lowest score received by a study was 37% [52], which was the result of using just one model, as well as the inadequate disclosure regarding sensitisation and sample sizes. While the study used TEM, Western blot and dynamic light scattering to profile the EVs, the lack of appropriate controls meant the study also received a low characterisation score. The highest score achieved by a study was 53% [51]. This study utilised multiple models and had good model robustness, as well as reasonable sample sizes. While using multiple techniques for EV characterisation, the lack of suitable characterisation controls resulted in a low score. All scores are outlined in Table 8.

## 4. Discussion

Allergies are a significant global issue with their prevalence and burden continuing to increase [1,2,53]. Despite this, the underlying mechanisms leading to the development of allergies are poorly understood. The recent and ever-growing research into extracellular vesicles provides a new avenue to further understand these mechanisms. In this systematic review, we analyse the current research suggesting the potential roles of epithelial-derived extracellular vesicles in facilitating allergic sensitisation or tolerance.

A total of 18 publications were identified that indicate that epithelial-derived EVs can cause effects with the potential to promote tolerance or allergic sensitisation. Some of the studies identified indicate that epithelial-derived EVs are able to drive sensitisation through the altered expression of miRNAs delivered to immune cells, ultimately facilitating the differentiation and polarisation of T cells into Th2 cells, as well as promoting Th2 cytokine secretion [37,38,39,40,41,47,49]. Yet in other studies, epithelial-derived EVs were shown to induce tolerance, promoting Treg and Tr1 differentiation by inducing the secretion of the cytokines TGF-β and IL-10 and suppressing the secretion of Th2 cytokines IL-5 and IL-13 [42,43,50,51,52]. This review, therefore, highlights that epithelial EVs can either promote tolerance or sensitisation depending on which epithelial barrier they are derived from and the status of the patient, cell or animal model used.

Regarding potential mechanisms, the studies suggest a number could be at play. One key mechanism identified is that ne-EVs are capable of promoting the Th2 differentiation of naïve CD4+ T cells, achieving this either directly or indirectly [37,39,41]. For example, a reduced expression of the miRNA146a-5p observed in allergic patients was shown to prevent the downregulation of Smad3/GATA-3 [41] as well as prevent the induction of Th2-suppressing IL-10+ monocytes [37], ultimately facilitating Th2 polarisation and allergic sensitisation [54,55]. Furthermore, upregulated lncGAS5 in allergic patients’ ne-EVs was shown to downregulate EZH2 expression in CD4+ cells, promoting Th2 differentiation as well as the production of Th2 cytokines, such IL-4, and sensitisation [39]. In contrast, another mechanism identified was the promotion of Th2 differentiation via DCs [38,47,49]. A pathway analysis suggested that the observed downregulation of miR34a, miR92b and miR210, as well as the downregulation of Lyn, could play an important role in the early development of allergy by promoting Th2 polarisation [38,47,56]. CNTN1-bearing bae-EVs were also shown to activate DCs, as well as upregulate the expression of CD40, again promoting Th2 differentiation. The current understanding of how the polarisation of naïve T cells is induced is incomplete [57]; however, the studies reviewed here suggest that EVs released from the epithelium can as act as a signal to further promote Th2 differentiation. Another method by which epithelial EVs can influence sensitisation is though the recruitment and induction of IL-6-, TNF-a- and IL-1β-secreting macrophages, promoting type 2 inflammation [44,45,48]. Ne-EVs were also shown to induce barrier damage and dysfunction as another mechanism for promoting sensitisation [35,36,40], an example being the finding that EVs containing lncRNA NEAT1 were also able to damage the epithelial barrier through the promotion of IL-13 secretion and apoptosis [40]. Epithelial barrier dysfunction can play a major role in facilitating increased allergen penetration and immune cell exposure, promoting sensitisation [58]. A study by Kortekaas et al. demonstrated that the maintenance of the nasal epithelial barrier was essential in the prevention of allergic sensitisation [59]. This further emphasises the role that epithelial EVs can play in the development of allergy.

As previously stated, epithelial-derived EVs are also capable of promoting immune tolerance. In particular, it is worth noting that, despite the small number of papers, all three identified studies on ie-EVs reported a tolerising influence and identified similar mechanisms through which ie-EVs can induce tolerance. All three studies suggest that OVA-induced ie-EVs facilitate the induction of Tr1 and Treg responses, suppressing Th2 responses and ultimately resulting in tolerance to the allergen [50,51,52]. One mechanism suggested that ie-EVs generated from OVA stimulation had an upregulated expression of αvβ6, which stimulated TGF-β secretion in DCs, suppressing sensitisation [50]. Furthermore, it was suggested that ie-EVs generated from an OVA challenge can also activate macrophages to secrete Treg polarising cytokines IL-10+ and TGF-β, further promoting Treg differentiation and tolerance [52]. Additionally, ie-EVs carrying IL-10, as well as MHC II/OVA peptide complexes, were shown to be able to recognise and trigger the differentiation of OVA-specific CD4+ T cells into Tr1 cells [51]. The role of the intestinal epithelium in the generation of oral tolerance, as well as the central role played by Tregs and other T regulatory cell subsets, is well established [60,61,62]. A study by Mucida et al. demonstrated the necessity of peripheral-induced antigen-specific Treg cells for oral tolerance [63], highlighting the importance of ie-EVs in the suppression of sensitisation. Another mechanism for the suppression of Th2 responses was identified in bae-EVS [42,43]. One study reported the induction of exosomes in Ole e 1-tolerised mice was shown to have a prophylactic effect on the sensitisation and challenge of naïve mice, inhibiting the production of an IgE response, as well as the production of Th2 cytokines [42]. This prophylactic effect was shown to also suppress sensitisation to an unrelated allergen Bet v 1 through the suppression of IL-5 and 13, suggesting EVs can induce ‘bystander tolerance’ [43].

It is important to draw attention to the generally low-quality scores attained by the studies in this review. These low scores can mainly be attributed to poor EV isolation methods. The studies concerning intestinal EVs had an average score of 47%, which was 14 points lower than the studies looking at ne-EVs, which had the next lowest (with an average score of 61%), potentially questioning the reliability of the mechanisms identified. The highest-scoring section was the bae-EV studies, which had an average score of 62%, which was only one point higher than the nasal studies, suggesting better reliability and robustness in the mechanisms identified in both these sections. This highlights the need for further high-quality studies investigating ie-EVs. The quality of EV isolation scores also emphasises the need for the implementation of more robust isolation methods that ensure better purity without the risk of EV damage or rupturing. Of the 18 studies identified, 72% of the studies received the minimum score for EV isolation [35,36,37,38,39,41,42,43,45,48,49,50,51], with only three studies obtaining the maximum score [40,46,47]. The studies had better scores for characterisation than isolation, with only 16% of the papers receiving the minimum possible characterisation score [35,36,50], while 28% received the maximum characterisation score [41–44,47). Moving forward, future research should employ techniques that minimize damage to EVs while ensuring high-purity isolation. Additionally, it is crucial to exclude other potential mediators, such as cytokines, which could obscure the downstream effects of EVs. In addition to this, the use of more comprehensive characterisation would allow for better identification and differentiation of EV sub-populations, allowing for more robust investigations into the different signals released by epithelial cells as a part of allergic sensitisation.

The robustness of model scores was significantly higher, with 50% of the studies using samples from human participants. Additionally, 67% of these studies received the maximum score for characterising allergic participants, identifying them from a clinical setting, and performing skin prick tests and serum IgE level tests [36,37,39,41,47,48]. The use of human samples gives a much more biologically relevant model for furthering the understanding of allergic sensitisation. A total of 67% of the papers utilised animal models (mouse models) [37,41,42,43,44,45,46,48,49,50,51,52]. Notable differences in human relevance include IL-10 being induced in both Th1 and Th2 responses in humans, but only induced in Th2 responses in mice. Additionally, mice express only CD1d receptors on dendritic cells, whereas humans express CD1a, b, c, d and e. Furthermore, murine models require artificial methods to induce allergies [64,65,66]. Lastly, despite good model robustness scores, no study performed any endotoxin measurement to ensure their sensitisation material was endotoxin-free. Endotoxins have been shown to promote inflammatory responses when exposed simultaneously with allergen [67,68]. Providing measurements of the endotoxin levels in the allergen preps used would provide further credibility to observed downstream immune responses.

Moving forward, EVs provide an exciting and rapidly growing area of research for further understanding the mechanisms through which epithelial cells influence allergic outcomes. It is evident from the studies identified in this review that EVs can play a role in both the induction of sensitisation and tolerance to allergens, and this role is affected by the epithelial barrier of origin, as well as changes in EV cargo as a result of patient health status and allergen challenge. More research into epithelial-derived EVs and the changes to cargo associated with allergen exposure is still needed to create a more comprehensive understanding of how epithelial-derived EVs can influence allergic sensitisation and tolerance. In addition, there was a notable gap in the current literature with a lack of articles studying the effect of EVs derived from the skin epithelium. The skin is the largest organ and is the first barrier to interact with environmental stimuli and consequently is a location where sensitisation can occur [69]. Furthermore, skin epithelial–immune cross-talk plays a role in allergic sensitisation and responses, secreting cytokines such as TSLP to influence Th2 polarisation [70,71]. Currently, there is a lot of research focusing on the role of EVs in the development of skin allergy and atopic dermatitis; however, these all focus on the effect of EVs derived from the skin microbiome or from immune cells rather than from the skin epithelium itself [33,72,73]. Future research on the role of skin epithelial-derived EVs in allergic sensitisation could uncover new mechanisms and potential therapeutic interventions. Finally, only one of the identified articles used a 3D cell culture model [47]. The adoption of more biologically relevant human cell culture models, such as air liquid interface (ALI) culture, would further enhance model robustness and provide a more biologically relevant model for investigating the role of epithelial-derived EVs in allergic sensitisation. As these techniques become more widely adopted, the evaluation of these models, as well as the mechanisms through which they are stimulated, could provide a more appropriate way to differentiate and score article robustness.

In conclusion, this systematic review provides some evidence of the effects that epithelial-derived extracellular vesicles can have on immune responses to allergens. The studies identified here suggest that epithelial EVs could play a pivotal role in influencing the allergic outcome via both direct and indirect mechanisms. The mechanisms identified that facilitate allergic sensitisation include the following: the promotion of naïve CD4+ T cell Th2 differentiation, the promotion of Th2 cells via DCs, the recruitment and induction of IL-6-, TNF-a- and IL-1β-secreting macrophages, and the induction of barrier damage and dysfunction. Conversely, some mechanisms were identified that showed epithelial-derived EVs promote allergic tolerance through the induction of Tr1 and Treg responses. The limited number of published studies and the lack of research on the skin epithelium highlight the need for dedicated research in these areas to enhance our understanding of epithelial-derived EVs and their role in promoting allergies.

## Figures and Tables

**Table 1:** The inclusion and exclusion criteria used to determine eligibility.

Inclusion	Exclusion
IgE-mediated allergy	Non-original research paper, e.g., reviews, commentary, case report, etc.
Epithelial-derived EVs	Non-IgE-mediated allergies
Allergic sensitisation	Non-English-language publications
Clinical data	Conference abstract
Experimental data	Research involving nanoparticles but not extracellular vesicles from a cell source
Healthy subjects	Pre-prints
Allergic subjects	Correspondence
Human studies	
Animal models	
Research involving the extraction, identification or production of EVs or their contents, such as DNA, miRNA or protein	
Isolation method of EVs included	

**Table 2:** Criteria used to derive study quality scores.

Category	Scoring Criteria
**Model:** If multiple models are used, a combined score is given	- Cell culture (in vitro—murine) (1); - Animal model (in vivo) (2); - Cell culture (in vitro—human) (3); - Human (ex vivo) (4).
**Material Used for Sensitisation:** Summative score of all points	- Allergen used to sensitize clearly defined (1); - Endotoxin measurements performed (1); - Protein content measurements performed (1).
**Robustness of Model:** If multiple models used a combined score is given	Human Studies (Ex Vivo): - Allergic status not specified or not clearly defined (0); - Allergic patients were sought from a clinical setting (1); - Allergic patients were sought from clinical setting AND had a positive test to allergen (specific IgE, skin test or challenge) (2); - Allergic patients were sought from a clinical setting with a non-sensitised control group (3); - Allergic patients were sought from clinical setting AND had a positive test to allergen (specific IgE or positive allergen challenge) with a healthy control group (4). Animal Model (In Vivo): - Sensitisation of animals not specified or defined (0); - Sensitisation of animals partially defined (discloses some but NOT all the following: exposure method, dosing and duration) (1); - Sensitisation of animals fully defined outlining exposure method, dosing and duration (2); - Sensitisation of animals partially defined (discloses some but NOT all the following: exposure method, dosing and duration) with a negative control group (3); - Sensitisation of animals fully defined outlining exposure method, dosing and duration with a negative control group (4). Cell Culture (In Vitro): - Transformed cell line using partially defined exposures (discloses some but NOT all the following: exposure method, dosing and duration) (1); - Transformed cell line using fully defined exposures (exposure method, dosing and duration) (2); - Primary cells using partially defined exposures (discloses some but NOT all the following: exposure method, dosing and duration) (3); - Primary cells using fully defined exposures (exposure method, dosing and duration) (4).
**Sample Size:** If multiple models used a combined score is given:	Human Studies (Ex Vivo): - Number of participants not defined (0); - Five or fewer participants per group (1); - Six to ten participants per group (2); - Eleven or more participants per group (3). Murine Model (In Vivo): - Number of animals per group not defined (0); - Five or fewer animals per group (1); - Six to ten animals per group (2); - Eleven or more animals per group (3). Cell Culture (In Vitro): - n not specified (0); - *n* = 1 (1); - *n* = 3 (2); - *n* > 3 (3).
**EV Isolation**	No Isolation—Precipitation-only techniques. Studies looking directly at a liquid with no isolation applied (usually performed using samples with very low volumes) (0); - Poor—Ultra-centrifugation at one speed or serial UC without sucrose cushion (1); - Fair—Size-exclusion chromatography (without a precipitation or concentration step) or immuno-capture beads for investigation of non-specific populations (2); - Good—Size-exclusion chromatography with a precipitation or concentration step or the use of a specific isolation method, such as exosome EV isolation, purification kits or immuno-capture beads utilising a unique marker (3);
**EV Characterisation**	No Characterisation—No attempt made to profile or characterise EVs or exosomes (0); - Poor—Use of just one characterisation technique (quantification, sizing, biomarkers or cargo analysis) (1); - Fair—The use of multiple complimentary characterisation techniques and at least 1 biomarker (2); - Good—Everything mentioned above as well as appropriate controls (e.g., robust profiling of culture conditions such as media and inclusion of positive and negative controls such as those recommended by MISEV2018) (3); - Very Good—Everything mentioned above as well the addition of extra biomarkers (3+) or the utilisation of any other novel techniques (4).

**Table 3:** Studies involving nasal epithelial-derived EVs (ne-EVs).

First Author and Year	Title	Allergen	Cells Responding	Model	Isolation	Characterisation	Reported Findings
Qiu et al., 2011 and 2012 [35,36]	**2011** Cytotoxic T lymphocytes mediate chronic inflammation of the nasal mucosa of patients with atypical allergic rhinitis [35] **2012** Antigen-specific activities of CD8+T cells in the nasal mucosa of patients with nasal allergy [36]	Der P 1	Dendritic cells and CD8+ T cells	Cell line (RPMI2650) and human patients	Serial Ultra-centrifugation: 300× *g* 10 min, 1200× *g* 20 min, 10,000× *g* 30 min, 100,000× *g* 1 h.	EM, Western blot and Bradford assay	Staphylococcal enterotoxin B (SEB) and Der p 1 containing EVs induced dendritic cell maturation, and generation of allergen specific granzyme B and perforin secreting CD8+ cytotoxic T Cells [35]. A higher frequency of CD8+ T cells in patient samples compared to controls [36].
Luo et al., 2015 [37]	Epithelial cell-derived microRNA-146a generates interleukin-10-producing monocytes to inhibit nasal allergy	N/A	Monocytes	Human patients, cell line (RPMI 2650) and mouse model (BALB/c)	Serial Ultra-centrifugation: 300× *g* 10 min, 1200× *g* 20 min, 10,000× *g* 30 min, 100,000× *g* 1 h.	Western blot and RTq-PCR	Reduced expression of mRNA-146α in EVs of patients with AR compared to healthy controls prevents the induction of IL-10^+^ monocytes and was shown to have a suppressive effect on CD4^+^ effector T cells and Th2 polarisation.
Wu et al., 2015 [38]	Altered microRNA Expression Profiles of Extracellular Vesicles in Nasal Mucus from Patients with Allergic Rhinitis	N/A	N/A	Human patients	Serial Ultra-centrifugation: 3000× *g* 15 min, 10,000× *g* 30 min, 50,000× *g* 1 h, 100,000× *g* 1 h.	FACS and RTq-PCR	Cargo analysis of EVs from patients with AR compared to healthy controls showed changes in expression of a wide variety of mRNAs involved in key pathways associated with allergic development, such as the c-fos, Lyn and MUC7
Zhu et al., 2020 [39]	Exosomal long non-coding RNA GAS5 suppresses Th1 differentiation and promotes Th2 differentiation via downregulating EZH2 and T-bet in allergic rhinitis	Ovalbumin	Naïve CD4+ T cells	Human patients and cell culture (RPMI 2650)	Serial Ultra-centrifugation: 12,000× *g* 45 min, 110,000× *g* 2 h, 110,000× *g* 70 min	TEM, Western blot and RTq-PCR	GAS5 can influence Th1/Th2 differentiation, downregulating T-bet and ultimately suppressing Th1 differentiation and promoting Th2 polarisation
Wang et al., 2021 [40]	Exosomal lncRNA Nuclear Paraspeckle Assembly Transcript 1 (NEAT1)contributes to the progression of allergic rhinitis via modulating microRNA-511/Nuclear Receptor Subfamily 4 Group A Member 2 (NR4A2) axis	N/A	N/A	Human patients and cell culture (primary cells)	Precipitation—EXOQuick Kit	TEM, Western blot and RTq-PCR	EVs containing LncRNA NEAT1 induce IL-13-mediated inflammatory responses and nasal epithelial cell apoptosis.
Li et al., 2023 [41]	ESP-B4 promotes nasal epithelial cell-derived extracellular vesicles containing miR-146a-5p to modulate Smad3/GATA-3 thus relieving allergic rhinitis ESP-B4/miR-146a-5p in AR	Ovalbumin	Naïve CD4+ T cells	Human patients, rat model (Wistar rats) and cell culture (RPMI2650)	Serial Ultra-centrifugation: 12,000× *g* 45 min, 110,000× *g* 2 h, 110,000× *g* 70 min	TEM, Western blot, NTA and RTq-PCR	Downregulation of miR-146α-5p in AR patients compared to healthy controls was shown to play an important role in Th1/Th2 differentiation

**Table 4:** Scoring of papers involving nasal epithelial-derived EVs (ne-EVs).

Author	Model (n/10)	Robustness of Model			Sample Size			Sensitisation Material (n/3)	EV Isolation (n/3)	EV Characterisation (n/4)	Total Score	Bias Score
		Murine Model (n/4)	Cell Culture (n/4)	Human Studies (n/4)	Model (n/3)	Cell Culture (n/3)	Human Studies (n/3)					
Qiu et al., 2011 [35]	Human patient samples (4) Cell culture—human (3)		Immortalized cell line, partially defined exposure (1)	From clinical setting and positive skin prick test (2)		3 repeats (2)	11 or more participants per group (3)	Allergen defined (1)	Serial UC (1)	One method utilized (1)	18/34	53%
Qiu et al., 2012 [36]	Human patient samples (4) Cell culture—human (3)		Immortalized cell line, partially defined exposure (1)	Allergic patients from clinical setting with serum IgE, IgG, skin prick test and non-allergic but chronic rhinitis controls (4)		3 repeats (2)	6–10 participants per group (2)	Allergen defined (1)	Serial UC (1)	One method utilized (1)	19/34	56%
Luo et al., 2015 [37]	Human patient samples (4), Cell culture—human (3), Animal model (2)	Sensitisation fully defined (4)	Immortalized cell line, partially defined exposure (1)	Allergic patients from clinical setting with serum IgE, IgG, skin prick test and healthy controls (4)	6–10 animals per group (2)	3 repeats (2)	6–10 participants per group (2)	Allergen defined (1)	Serial UC (1)	Multiple complimentary techniques (2)	28/41	66%
Wu et al., 2015 [38]	Human patient samples (4)			Allergic patients from clinical setting with serum IgE, IgG, skin prick test and healthy controls (4)			11 or more participants per group (3)		Serial UC (1)	Multiple complimentary techniques (2)	14/27	52%
Zhu et al., 2020 [39]	Human patient samples (4)			Allergic patients from clinical setting with serum IgE, IgG, skin prick test and healthy controls (4)			11 or more participants per group (3)		Serial UC (1)	Multiple complementary techniques and suitable controls (3)	15/27	56%
Wang et al., 2021 [40]	Human patient samples (4) Cell culture—human (3)		Primary cell line and fully defined exposures (4)	Allergic patients from a clinical setting and a control group (3)		3 repeats (2)	11 or more participants per group (3)		Precipitation—EXOQuick-TC (3)	Multiple complementary techniques and suitable controls (3)	25/34	74%
Li et al., 2023 [41]	Human patient samples (4), Cell culture—human (3), Animal model (2)	Sensitisation fully defined (4)	Transformed cell line and fully defined exposure (2)	Allergic patients from clinical setting with serum IgE, IgG, skin prick test and healthy controls (4)	6–10 animals per group (2)	3 repeats (2)	11 or more participants per group (3)	Allergen defined (1)	Serial UC (1)	Multiple complementary techniques, suitable controls and additional biomarkers (4)	32/41	78%

**Table 5:** Studies involving airway epithelial-derived EVs.

First Author and Year	Title	Allergen	Cells Responding	Model	Isolation	Characterisation	Outcome
Prado et al., 2008 [42]	Exosomes from bronchoalveolar fluid of tolerized mice prevent allergic reaction	Ole e 1	T Cells	Mouse model (BALB/c) and cell culture	Ultracentrifugation: 100,000× *g* for 18 h	EM, Western blot and FACS	EVs isolated from the BALF of mice tolerised to the allergen Ole e 1 were able to inhibit Th2 responses, suppressing IgE and IgG1 and upregulating TGF-β
Prado et al., 2010 [43]	Bystander suppression to unrelated allergen sensitisation through intranasal administration of tolerogenic exosomes in mouse	Ole e 1 and Bet v 1	T Cells	Mouse model (BALB/c) and cell culture	Ultracentrifugation: 100,000× *g* for 18 h	EM, Western blot and FACS	Bystander suppression was observed after nasal administration of BALF-derived EVs from tolerised mice inhibiting sensitisation to other allergens, supressing IgE and IgG1 as well as the Th2 cytokines Il-5 and IL-13
Shin et al., 2010 [44]	Extracellular vesicles are key intercellular mediators in the development of immune dysfunction to allergens in the airways	Ovalbumin	T Cells and dendritic cells	Mouse model (BALB/c)	Sucrose Cushion Serial Ultracentrifugation: 500× *g* 10 min, 3000× *g* 20 min, Cushioned: 100,000× *g* 2 h 100,000× *g* 2 h.	TEM, Western blot, FACS and Bradford assay	LPS exposure enhanced airway epithelial derived-EV (ae-EV) production. These LPS-induced EVs were shown to enhanced sensitisation to allergens and promote TNF-a and IL-6 secretion in macrophages
Kulshreshtha et al., 2013 [45]	Proinflammatory role of epithelial cell-derived exosomes in allergic airway inflammation	Ovalbumin	Monocytes	Mouse model (BALB/c) cell culture (BEAS-2B)	Serial Ultracentrifugation: 300× *g* 5 min, 800× *g* 5 min, 2000× *g* 10 min, 10,000× *g* 30 min, 70,000× *g* 60 min	TEM, Western blot and FACS	Th2 cytokine-stimulated epithelial cells had increased EV secretion and cargo changes. These EVs induce monocyte proliferation
Gon et al., 2017 [46]	Selective release of miRNAs via extracellular vesicles is associated with house-dust mite allergen-induced airway inflammation	House dust mite	CD4+ T helper cells	Mouse model (C57BL/6J)	Precipitation—EXOQuick Kit	TEM, Western blot and qNano counter	EVs used to remove Th2 inhibitory miRNAs that downregulate IL-5 and Il-13 receptors on epithelial cells
Bartel et al., 2020 [47]	Human airway epithelial extracellular vesicle miRNA signature is altered upon asthma development	N/A	N/A	Human patients and cell cell culture (primary cells)	Precipitation—EXOQuick Kit	TEM, Western blot, NTA and SeramiR miRNA	Changes in expression of miR-34a, miR-92b and miR-210 predicted by pathway analysis to promote DC-induced Th2 polarisation of CD4+ T cells, regulating Th2 polarisation and DC maturation
Yu et al., 2021 [48]	Increased airway epithelial cell-derived exosomes activate macrophage-mediated allergic inflammation via CD100 shedding	Ovalbumin	macrophages	Mouse models (C57BL/6J) and cell culture (primary cells and BEAS-2B)	Serial Ultracentrifugation: 300× *g* 10 min, 3000× *g* 15 min, 10,000× *g* 30 min 100,000× *g* 70 min, 100,000× *g* 70 min.	TEM, Western blot and NTA	OVA containing EVs promote infiltration of neutrophils, monocytes and DCs into the lung and induce macrophages to secrete IL-6, TNF-a and IL-1β
Zhang et al., 2021 [49]	Epithelial exosomal contactin-1 promotes monocyte-derived dendritic cell-dominant T-cell responses in asthma	House dust mite	Dendritic cells	Human patient, mouse model (C57BL/6N) and cell culture (primary cells)	Serial Ultracentrifugation: 2000× *g* 10 min, 10,000× *g* 30 min 100,000× *g* 70 min, 100,000× *g* 70 min.	TEM, Western blot and NTA	HDM stimulation released EVs that recruited DCs in the lung. These EVs can activate DC though the cargo CNTN1 and upregulate the expression of CD40

**Table 6:** Scoring of papers involving airway epithelial-derived EVs.

Author	Model (n/10)	Robustness of Model			Sample Size			Sensitisation Material (n/3)	EV Isolation (n/3)	EV Characterisation (n/4)	Total Score	Bias Score
		Murine Model (n/4)	Cell Culture (n/4)	Human Studies (n/4)	Murine Model (n/3)	Cell Culture (n/3)	Human Studies (n/3)					
Prado et al., 2008 [42]	Animal model (2), cell culture—murine (1)	Sensitisation fully defined (4)	Primary cell line and fully defined exposure (4)		11 or more mice per group (3)	more than 3 repeats (3)		Allergen defined (1)	UC (1)	Multiple complementary techniques, suitable controls and additional biomarkers (4)	23/34	68%
Prado et al., 2010 [43]	Animal model (2), cell culture—murine (1)	Sensitisation fully defined (4)	Primary cell line and fully defined exposure (4)		5 or fewer mice per group (1)	more than 3 repeats (3)		Allergen defined (1)	Serial UC (1)	Multiple complementary techniques, suitable controls and additional biomarkers (4)	21/34	62%
Shin et al., 2010 [44]	Animal model (2)	Sensitisation fully defined (4			5 or fewer mice per group (1)			Allergen defined (1)	Sucrose Cushioned UC (2)	Multiple complementary techniques, suitable controls and additional biomarkers (4)	14/27	52%
Kulshreshtha et al., 2013 [45]	Cell culture—human (3), animal model (2)	Sensitisation fully defined (4)	Transformed cell line and partially defined exposure (1)		6–10 mice per group (2)	3 repeats (2)		Allergen defined (1)	Serial UC (1)	Multiple complementary techniques and suitable controls (3)	19/34	56%
Gon et al., 2017 [46]	Animal model (2)	Sensitisation fully defined (4)			11 or more mice per group (3)			Allergen defined (1)	Precipitation—EXOQuick-TC (3)	Multiple complementary techniques and suitable controls (3)	16/27	59%
Bartel et al., 2020 [47]	Human patient samples (4), cell culture—human (3)		Primary cell line and fully defined exposure (4)	Allergic patients from clinical setting with serum IgE, IgG, skin prick test and healthy controls (4)		3 repeats (2)	6–10 participants per group (3)		Precipitation—EXOQuick-TC (3)	Multiple complementary techniques, suitable controls and additional biomarkers (4)	27/34	79%
Yu et al., 2021 [48]	cell culture—human (3), animal model (2)	Sensitisation fully defined (4)	Immortalised cell line and fully defined exposure (2)		5 or fewer mice per group (1)	Not specified (0)		Allergen defined (1)	Serial UC (1)	Multiple complementary techniques and suitable controls (3)	17/34	50%
Zhang et al., 2021 [49]	Human patient samples (4), animal model (2) cell culture—murine (1)	Sensitisation fully defined (4)	Primary cell line and partially defined exposures (3)	Allergic patients from a clinical setting and a control group (3)	5 or fewer mice per group (1)	Not specified (0)	6–10 participants per group (3)	Allergen defined (1)	Serial UC (1)	Multiple complementary techniques and suitable controls (3)	26/41	63%

**Table 7:** Studies involving intestinal epithelial-derived EVs.

First Author	Title	Allergen	Cells Responding	Model	Isolation	Characterisation	Outcome
Chen et al., 2011 [50]	Intestinal epithelial cell-derived integrin αβ6 plays an important role in the induction of regulatory T cells and inhibits an antigen-specific Th2 response	Ovalbumin	Dendritic cells	Mouse model (Balb/c); cell culture (IEC4.1)	Serial Ultracentrifugation: 300× *g* 10 min, 1200× *g* 20 min, 10,000× *g* 30 min, 100,000× *g* 1 h	EM, Western blot and Bradford assay	Intestinal epithelial cells post-OVA uptake secrete EVs containing integrin αvβ6 and OVA. These EVs induced antigen-specific Tregs and TGF-β+ DCs
Zeng et al., 2020 [51]	Exosomes carry IL-10 and antigen/MHC II complexes to induce antigen-specific oral tolerance	Ovalbumin	Tregs and Tr1 Cells	Cell culture (mode K cells) and mouse models (VIPd and BALB/c)	Serial Ultracentrifugation: 300× *g* 10 min, 1200× *g* 20 min, 10,000× *g* 30 min, 100,000× *g* 1 h	Western blot and Bradford assay	VIPd mice fail to induce Tr1 cells in the intestine. EVs from OVA/VIP-primed IECs carry allergen-MHC II complexes and IL-10, which are able to induce Tr1 differentiation in OVA-specific CD4^+^ cells; the administration of these suppressed experimental food allergy.
Shin et al., 2022 [52]	Extracellular vesicles derived from small intestinal lamina propria reduce antigen-specific immune response	Ovalbumin	Tregs	Mouse model (C57BL/6)	Sucrose Cushion Serial Ultracentrifugation: 500× *g* 10 min, 3000× *g* 20 min, Cushioned: 100,000× *g* 2 h 100,000× *g* 2 h.	TEM, Western blot, Bradford assay and dynamic light scattering (sizing)	EVs containing OVA and MHCII induce CD4^+^Foxp3^+^ T cell differentiation and promote the secretion of Treg-promoting cytokines IL-10 and TGF-β in macrophages

**Table 8:** Scoring of papers outlining intestinal epithelial-derived EVs.

Author	Model (n/10)	Robustness of Model			Sample Size			Sensitisation Material (n/3)	EV Isolation (n/3)	EV Characterisation (n/4)	Total Score	Bias Score
		Murine Model (n/4)	Cell Culture (n/4)	Human Studies (n/4)	Murine Model (n/3)	Cell Culture (n/3)	Human Studies (n/3)					
Chen et al., 2011 [50]	Animal model (2), cell culture—murine (1)	Sensitisation fully defined (4)	Transformed cell line and fully defined exposure (2)		6–10 mice per group (2)	3 repeats (2)		Allergen defined (1)	Serial UC (1)	Multiple complimentary techniques (2)	17/34	50%
Zeng et al., 2020 [51]	Animal model (2), cell culture—murine (1)	Sensitisation fully defined (4)	Transformed cell line and fully defined exposure (2)		6–10 mice per group (2)	more than 3 repeats (3)		Allergen defined (1)	Serial UC (1)	Multiple complimentary techniques (2)	18/34	53%
Shin et al., 2022 [52]	Animal model (2)	Sensitisation of mice partially defined (3)			Number not defined (0)			Allergen defined (1)	Sucrose Cushioned UC (2)	Multiple complimentary techniques (2)	10/27	37%

## Data Availability

The original contributions presented in the study are included in the article; further inquiries can be directed to the corresponding author.

## Supplementary Materials

The following supporting information can be downloaded at: https://www.mdpi.com/article/10.3390/ijms26125791/s1.

## Abbreviations

The following abbreviations are used in this manuscript:

EV	Extracellular Vesicle
DC	Dendritic Cells
ne-EVs	Nasal Epithelial-Derived Extracellular Vesicles
bae-EVs	Bronchial/Alveolar Epithelial-Derived Extracellular Vesicles
ie-EVs	Intestinal Epithelial-Derived Extracellular Vesicles
TJ	Tight Junction
IL	Interleukin
AR	Allergic Rhinitis
IgE	Immunoglobulin E
