# Supplementary material for: The Role of Epithelial-Derived Extracellular Vesicles in Allergic Sensitisation: A Systematic Review
Source: Int J Mol Sci. 2025 Jun 17;26(12):5791. doi: 10.3390/ijms26125791 (PMC12193434; doi:10.3390/ijms26125791)
Supplement: Supplementary file 1 [file ijms-26-05791-s001.zip › ijms-3676183-supplementary.pdf]

PubMed search terms:

((Epithelial OR Epithelium) AND (Extracellular Vesicles OR EVs OR microvesicles OR exosome OR Shedding Vesicle) AND (Skin OR lung OR gut OR Oral Or Nasal) AND (sensitisation OR sensitization OR allergic response Or allergy OR Inflammation OR type 1 Hypersensitivity OR IgE OR Toler*))) NOT review)

Embase Search terms:

((Epithelial OR Epithelium OR BALF OR Bronchoalveolar lavage Fluid OR Lavage) AND (Extracellular Vesicles OR EVs OR microvesicle* OR exosome* OR ectosome OR Shedding vesicle OR Shedding vesicles OR Microparticle) AND (Skin OR lung OR gut OR Nasal OR Oral OR Muosa*) AND (allergic response OR allergy OR allergen OR sensitisation OR sensitization OR type 1 Hypersensitivity OR IgE OR Toler*))

Web of Science Search terms:

(((TS=(Epithelial OR Epithelium OR BALF OR Bronchoalveolar lavage Fluid OR Lavage) AND TS=(Extracellular Vesicles OR EVs OR microvesicle* OR exosome* OR ectosome OR Shedding vesicle OR Shedding vesicles OR Microparticle) AND TS=(Skin OR lung OR gut OR Nasal OR Oral OR Muosa*) AND TS=(allergic response OR allergy OR allergen OR sensitisation OR sensitization OR type 1 Hypersensitivity OR IgE OR Toler*)))) – web of science
